# Bladder Cancer Stem-Like Cells: Their Origin and Therapeutic Perspectives

**DOI:** 10.3390/ijms17010043

**Published:** 2015-12-29

**Authors:** Tomokazu Ohishi, Fumitaka Koga, Toshiro Migita

**Affiliations:** 1 Institute of Microbial Chemistry (BIKAKEN), Numazu, 18-24 Miyamoto, Numazu-shi, Shizuoka 410-0301, Japan; ohishit@bikaken.or.jp; 2 Division of Molecular Biotherapy, Japanese Foundation for Cancer Research, 3-8-31 Ariake, Koto-ku, Tokyo 135-8550, Japan; 3 Department of Urology, Tokyo Metropolitan Cancer and Infectious Diseases Center, Komagome Hospital, Tokyo 113-8677, Japan; f-koga@cick.jp; 4 Department of Urology, Kashiwa Kousei General Hospital, 617 Shikoda, Kashiwa-shi, Chiba 277-8551, Japan

**Keywords:** cancer stem cell, bladder cancer

## Abstract

Bladder cancer (BC), the most common cancer arising from the human urinary tract, consists of two major clinicopathological phenotypes: muscle-invasive bladder cancer (MIBC) and non-muscle-invasive bladder cancer (NMIBC). MIBC frequently metastasizes and is associated with an unfavorable prognosis. A certain proportion of patients with metastatic BC can achieve a remission with systemic chemotherapy; however, the disease relapses in most cases. Evidence suggests that MIBC comprises a small population of cancer stem cells (CSCs), which may be resistant to these treatments and may be able to form new tumors in the bladder or other organs. Therefore, the unambiguous identification of bladder CSCs and the development of targeted therapies are urgently needed. Nevertheless, it remains unclear where bladder CSCs originate and how they are generated. We review recent studies on bladder CSCs, specifically focusing on their proposed origin and the possible therapeutic options based on the CSC theory.

## 1. Introduction

Tissues can regenerate in the event of partial loss or damage. Stem cells are both pluripotent and have self-renewal potential, thereby contributing to tissue regeneration. Tumor cells are heterogeneous and are assumed to include stem cell-like cancer cells, or so-called cancer stem cells (CSCs). The first experimental evidence of CSCs was described in previous landmark studies in hematology [1,2]. According to the consensus definition of CSCs [3], these cells have the capacity for self-renewal and for generating heterogeneous lineages of cancer cells that compose the tumor. However, it is technically difficult to demonstrate the pluripotency and self-renewal potential of cancer cells *in vivo*, especially in solid tumors. Currently, the tumorigenicity of a small number of cancer cells in immunodeficient mice is used as the gold standard for the confirmation of CSCs. Thus, the term CSCs is considered to be a conceptual or empirical term. Tumor-initiating cells, tumor-propagating cells [4], or tumor-perpetuating cells [5] may be a more accurate term; however, we use the term CSCs to include cancer stem-like cells. CSCs were first identified in leukemia and were then found in solid tumors, such as breast [6], brain [7], colorectal [8,9,10], head and neck [11], pancreatic [12], and prostate cancers [13], as well as melanoma [14].

Bladder cancer (BC), which originates from the urothelial epithelium, is the most common cancer of the human urinary tract. BC is the ninth most common cancer worldwide and is relatively common in developed countries [15]. Men have a higher incidence of BC than women (ratio 3.5:1) [15]. BC is divided into two clinicopathologic entities: non-muscle-invasive bladder cancer (NMIBC) and muscle-invasive bladder cancer (MIBC). Approximately 80% of BC patients present with NMIBC, which is associated with a lower risk of mortality despite the high risk of intravesical tumor recurrence. In contrast, the remaining 20% of patients present with MIBC, which frequently metastasizes to other distant organs, including the liver, lung, and bone. Patients with MIBC have a higher risk of mortality. The pathological differences between NMIBC and MIBC are remarkable. Most cases of NMIBC are characterized by a papillary structure, whereas MIBC does not have such a uniform structural pattern. Compared with NMIBC, MIBC consists of a wider variety of cancer cells that range from differentiated to undifferentiated. Urothelial stem cells localize in the basal cell layer and can generate all types of urothelial cells [16,17,18,19]. When the urothelium is partially damaged or lost, urothelial stem cells can regenerate and compensate for the damaged or lost tissue. However, the regenerative potential of stem cells is well coordinated and strictly regulated in the normal urothelial lineage, thereby preventing tumor formation. BC cells are considered to arise from a single urothelial cell that has accumulated genetic or epigenetic DNA alterations. Bladder CSCs were first identified in 2009 by sorting with markers of normal basal cells [20]. Recent studies have demonstrated new approaches for identifying bladder CSCs, and the results of these studies increasingly suggest the existence of bladder CSCs [21,22,23,24,25,26,27]. However, the origin of bladder CSCs and the mechanism of their generation are largely unknown [21]. This review focuses on the putative origin of bladder CSCs and addresses the potential therapeutic approaches for advanced BC based on the CSC theory.

## 2. Tumor Heterogeneity and the Cancer Stem Cell (CSC) Theory

Tumors are believed to originate from a single cancer cell. It has long been discussed how this initial cancer cell generates other different types of cancer cells to form a tumor. Tumor heterogeneity may be caused by the heterogeneity of cancer cells at both the innate genetic and phenotypic levels. Because of genetic instability in cancer cells, random mutations accumulate during cell division, and the progeny may have distinct genotypes from the parental cancer cells. Genomic and epigenomic variations have been observed in each subpopulation of cancer cells within a single tumor [28,29,30]. During proliferation, each cancer cell encounters a hostile environment; therefore, cancer cells have to modify and adjust their phenotype according to the tumor microenvironment. Solid tumors less than 1–2 mm in diameter can obtain sufficient nutrition and oxygen from their surrounding environment, but the cells in the center of a larger solid tumor usually grow slower than those in the marginal region [31,32]. Accordingly, tumors are composed of various cancer cells that have distinct genomic, epigenomic, and phenotypic alterations, and tumor heterogeneity is an inherent and fundamental property of solid tumors.

Two major hypotheses may explain how tumors develop. In the stochastic model, all tumor cells have tumorigenic potential, and each heterogenic tumor cell can individually form tumors. In the hierarchy model, only a small population of CSCs have the ability to produce various progenitor tumor cells and to form tumors, but the non-CSCs cells do not have these properties. Recent studies support the hierarchy model, emphasizing the significance of CSCs, and propose evolutional models based on the CSC theory [33,34,35,36].

CSCs in tumors have several common properties, including a slower growth rate and transition to a dormant cell population compared with the majority of other tumor cells. This characteristic of CSCs has important clinical implications because it is associated with resistance to anticancer therapeutics [3,5,34,37]. Classical chemotherapy and radiotherapy cause less damage to DNA in slow-dividing cells, such as CSCs. In addition, CSCs display higher levels of drug efflux proteins, such as P-glycoproteins and ATP-binding cassette transporters [38]. Therefore, a causal relationship between CSCs and drug resistance has been suggested; however, the efflux mechanism is not likely to be crucial for CSCs [37]. Another common property of CSCs is high tumorigenicity. A relatively smaller number of CSCs can form tumors in xenograft models compared with non-CSCs. A single melanoma cell can form tumors, independent of CSCs markers [39]; therefore, the robustness of tumorigenicity varies among cell lines or with the method of CSC isolation. In the clinical setting, after significantly reducing the tumor burden by surgery, chemotherapy or radiation therapy, tumors can recur from a small number of residual CSCs. Additionally, high tumorigenicity is associated with the potential for tumor regrowth in other organs, known as distant metastasis.

**Figure 1:**
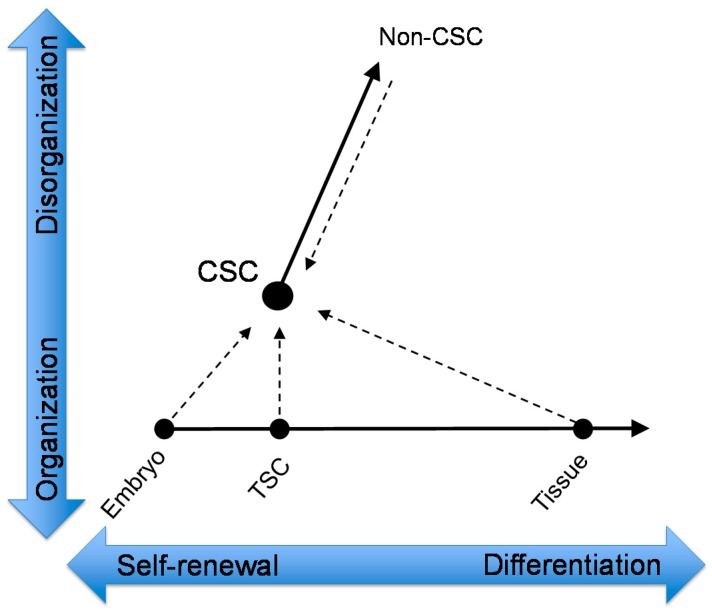
Schematic model of cancer stem cell (CSC) generation. The embryo undergoes a series of cleavage divisions and generates multipotent embryonic stem cells (ESCs). The ESCs generate tissue stem cells (TSCs), which are indispensable for tissue generation. The TSCs include urothelial stem cells, which produce all types of urothelial cells via differentiation. CSCs are separate from the normal differentiation process because of their lack of organization (dotted arrows). CSCs generate more differentiated cancer cells, known as non-CSCs.

Tissue stem cells originally develop from a single embryonic cell. The cellular differentiation of tissue stem cells is completely programmed in the DNA of the embryonic cell. On the other hand, cancer is a disease caused by cells that have lost the programming for terminal differentiation [40]. Thus, all cancer cells are considered to be undifferentiated cells, and CSCs are likely to be the most immature, undifferentiated cancer cells. Normal stem cells have a homeostatic ability to balance differentiation and self-renewal based on environmental stimuli and genetic constraints [41]; however, this balance in CSCs is inclined toward self-renewal because CSCs remain in an undifferentiated state. Moreover, cancer cells are immortal and grow indefinitely despite the growth-suppressing effects by surrounding normal cells, limited energy supply, and immunological surveillance. Cancer cell proliferation is not restricted by any type of host cell, which is partially caused by miscommunication between cancer cells and surrounding normal cells. Compared with hematologic CSCs, this characteristic of CSCs in solid tumors is critical because it allows them to form disorganized tumor tissues. Collectively, the developmental evolution of normal and cancer cells could be represented graphically by a relationship between their differentiation potential (horizontal axis) and organizing ability (vertical axis) (Figure 1). In this context, tumor-generating CSCs may arise from a type of normal or cancer cell, but CSCs are likely to be cells with high self-renewal potential and high organizing potential. Alternatively, non-CSCs may have self-renewal potential to some extent; however, they are unlikely to have tissue-organizing properties.

## 3. The Possible Existence of Bladder CSCs

Bladder tissue functions as a reservoir for urine and protects the host from urinary constituents. Urothelial cells including stem cells are usually quiescent; however, they will proliferate and stem cells supply new urothelial cells for the repair and reconstitution of bladder tissue when the bladder mucosa is damaged by various pathogens (mainly via infections) [42]. Bladder tissue includes many types of stem cells, such as urothelial stem cells, adipose-derived stem cells, bone marrow-derived mesenchymal stromal cells, mesenchymal stem cells, urine-derived stem cells [43,44]. BC may originate from such stem cells or from more differentiated progenitor cells as a result of genetic or epigenetic alterations. Recent molecular biology studies have demonstrated that BC results from specific genetic mutations, and such genetic variations corresponded well to the clinicopathological phenotype [45,46]. The occurrence of NMIBC and MIBC may depend on their originating site and aberrations in a specific signaling pathway. NMIBC exhibits frequent mutations of fibroblast growth factor receptor 3 (*FGFR3*), *HRAS*, and *PIK3CA* in differentiated (uroplakin^+^ and cytokeratin-20^+^) or intermediate (cytokeratin-18^+^, p63^+/−^, cytokeratin-5^+/−^, and CD44^+/−^) urothelial cells, whereas MIBC exhibits mutations of the tumor suppressor genes *p53*, *Rb*, and *PTEN* in basal cells (cytokeratin-5^+/−^, cytokeratin-17^+^, CD44^+/−^, and p63^+^) [22,23,47].

The molecular profiling of established BC cell lines has demonstrated distinct expression patterns between NMBIC and MIBC. A wide variety of stem cell markers are up-regulated in CSCs obtained from MIBC cell lines [48]. Importantly, most bladder CSCs have been identified in highly metastatic MIBC but not in NMIBC [20,49,50,51,52,53]. The majority of metastatic BCs initially respond to systemic chemotherapy, but metastatic lesions may subsequently appear despite the continuous administration of treatment. The existence of bladder CSCs may explain observations in the clinical setting, including the most important clinical issues: chemoresistance and metastasis.

The hierarchy model and the CSC theory are entirely dependent on the well-defined detection and verification of CSCs within a tumor. The following techniques have been developed to identify CSCs, including bladder CSCs: a side population method with DNA-binding Hoechst 33342 or DyeCycle Violet [48,50,51], aldehyde dehydrogenases (ALDH) activity [52,54], *in vitro* sphere formation [55,56], and CSC markers [22,24]. Currently, a flow cytometric technique with CSC markers is widely used to detect CSCs. CD44 is a member of the transmembrane glycoprotein family and has been implicated as a CSC marker in many malignancies, including head and neck [11], gastric [57], prostate [58], colorectal [10], and pancreatic cancer [12]. In BC, CD44^+^ cells exhibit an enhanced capacity to form xenografts in immune-compromised mice and exhibit chemoresistance compared with CD44^−^ cells [20,59]. CD44v6, a CD44 variant isoform containing the CD44v6 exon, has been shown to be enriched in bladder CSCs [53,60]. Other bladder CSC markers have been reported, including CD133 [61,62], 67-kDa laminin receptor (67LR) [49], CD47 [20], CD49 [63], and keratin 14 (*KRT14*) [64]. The sequential transplantation of xenograft tumors is increasingly required for the confirmation of CSCs [3,5,35]; however, studies on BC remain scarce. Collectively, the existence of bladder CSCs remains uncertain; however, it is evident that BC displays tumor heterogeneity, and certain BC cells have unique and advantageous properties for their survival and development.

## 4. Hypothetical Origin of Bladder CSCs

The discovery of induced pluripotent stem (iPS) cells has impacted stem cell biology and oncology. CSCs can be experimentally obtained through the generation of iPS cells [65]. Immature reprogrammed iPS cells can develop renal cancer in chimeric mice, which suggests that the epigenetic alterations of normal cells promotes the generation of tumor-initiating cancer cells [66]. Furthermore, the implantation of embryonic stem (ES) cells or iPS cells in mice results in teratoma formation and cancer [67,68]. In addition to iPS cells, human mesenchymal stem/stromal cells spontaneously transform after long-term culture [69]. These findings suggest that multipotent stem cells are likely to transform into CSCs, thereby resulting in tumorigenesis. However, importantly, mutations or chromosomal rearrangements in progenitor cells and differentiated cells as well as in stem cells, may give rise to CSCs [70]. Malignant transformation may result from the activation of the oncogenic pathway, independent of the cell-of-origin [71]. Thus, although stem cells are most likely to be the origin of CSCs, it cannot exclude the possibility of more differentiated epithelial cells as the origin of CSCs.

Another hypothesis for the origin of CSCs is that cancer cells may generate these cells on an autonomous basis. Increasing evidence of cancer cell plasticity between CSCs and non-CSCs [36,72] indicates that non-CSCs can generate CSCs. The overexpression of oncogenes *in vitro* can transform human fibroblasts into the CSC phenotype, including properties of self-renewal, multipotency, and the generation of heterogeneous tumors [73]. Pre-existing cancer cells have genetic instability; therefore, these cells easily acquire random mutations, chromatin modifications, and epigenetic reprogramming. The generation of iPS cells allows us to hypothesize that differentiated cancer cells could be reverted into CSCs by the activation of defined transcriptional factors [68]. Several reports have suggested that the phenotype of cancer cells transforms into that of CSCs when cells are transfected with the defined factors Oct3/4, Sox2, Klf4, and c-Myc [74].

Taken together, these results indicate that CSCs may originate from both normal cells and pre-existing cancer cells. In the next section, we discuss the possible origins of bladder CSCs.

### 4.1. Normal Urothelium

**Figure 2:**
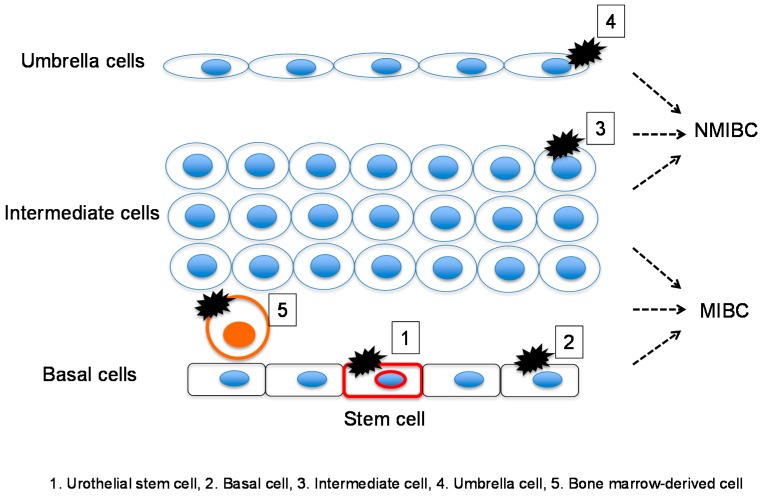
Normal bladder tissue may be a possible origin of bladder CSCs by direct transformation into CSCs via mutations in urothelial stem cells, basal cells, intermediate cells, and terminally differentiated umbrella cells. Urothelial stem cells, basal cells and intermediate cells give rise to muscle-invasive bladder cancer (MIBC), whereas umbrella and intermediate cells give rise to non-muscle-invasive bladder cancer (NMIBC).

The bladder urothelial mucosa is composed of three types of urothelial cells: basal, intermediate, and differentiated umbrella [16,17,18]. Importantly, a genetic mouse model for BC has demonstrated that BCs arise from these distinct urothelia [75]. McConkey’s group performed a clustering analysis of the gene expression profile of MIBC and demonstrated that this cancer can be further classified into basal, luminal, and *p53*-like subtypes based on site-specific molecular markers and resistance to chemotherapy [76]. Thus, bladder CSCs may originate from these intrinsic normal cells (Figure 2).

#### 4.1.1. Urothelial Stem Cell

Intestinal stem cells expressing Lgr5 are suggested to be the origin of colon CSCs [77]. This finding indicates that CSCs may directly originate from normal stem cells. A pulse-chase study with the nucleotide analogue bromodeoxyuridine demonstrated that cells with label-retaining DNA, which are considered to be urothelial stem cells, were localized to the basal cell layer [19]. Another study using mitochondrial DNA mutations supports this finding [44]. The nitrosamine-induced BC model suggests that MIBC originates from stem cells in the basal cell layer [78]. These results suggest that urothelial stem cells located in the basal cell layer are most likely the origin of bladder CSCs.

#### 4.1.2. Other Stem Cells in the Normal Urothelium

In addition to urothelial stem cells, bone marrow-derived stem cells, adipose-derived stem cells and urine-derived stem cells are used as sources for bladder repair [43]. Additionally, these stem cells are a possible origin of bladder CSCs. *Helicobacter pylori*, a carcinogen, causes gastric cancer through the recruitment of bone marrow-derived cells (BMDCs) [79]. However, in the chemical-induced BC model, BMDCs were associated with inflammation surrounding the tumor but not with tumorigenesis [80].

#### 4.1.3. Basal Cells

The first report of bladder CSCs demonstrated that these cells were enriched in basal cell markers (Lineage-CD44^+^CK5^+^CK20^−^) [20]. The putative markers of bladder CSCs are mostly overlapped with those of basal cells [25]. Furthermore, the gene expression meta-dataset demonstrated that the gene signature of basal-like MIBC is enriched for genes expressed by basal-like breast cancer and tumor-initiating cells [81]. Shin *et al.* reported that MIBC arises exclusively from Sonic hedgehog (Hh)-expressing basal cells *in vivo* [82]. Keratin-5-expressing basal cells give rise to carcinoma *in situ*, MIBC, and squamous cell carcinoma (SCC) [75]. These findings support the hypothesis that bladder CSCs originate from basal cells.

#### 4.1.4. Intermediate Cells

Intermediate cells show variable expression of a bladder CSC marker, CD44 [53]. The linage-tracing experiment in a murine carcinogenesis model showed that intermediate cells give rise primarily to papillary bladder tumors [75]. Additionally, Brandt *et al.*, suggested that the malignant transformation of intermediate cells by *FGFR3* expression leads to hyperplasia and low-grade papillary tumors [26]. These findings suggest that intermediate cells are a possible origin of CSCs in NMIBC.

#### 4.1.5. Umbrella Cells

Luminal-type MIBC may originate from umbrella cells via the aberrant expression of transcriptional factors, such as *PPARG*, *ESR1*, and *FGFR3* [76]. In addition, another report showed that luminal-typed MIBC expresses umbrella cell markers, such as uroplakins and low-molecular-weight keratin 20 [81]. Thus, MIBC may originate from umbrella cells, which may transform into bladder CSCs.

### 4.2. Bladder Cancer (BC) Cells

**Figure 3:**
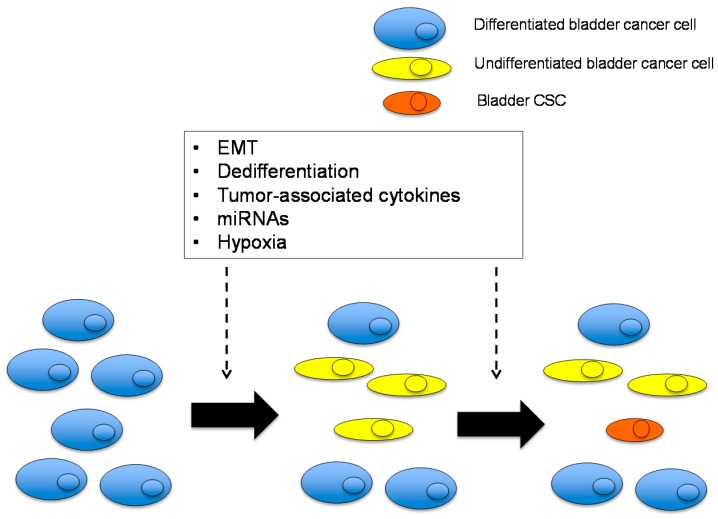
Possible mechanisms of bladder CSC generation from more differentiated bladder cancer (BC) cells. Differentiated and undifferentiated BC cells, which are defined as non-CSCs, are a possible origin of bladder CSCs. This phenomenon is caused by cell-autonomous mechanisms and the tumor microenvironment, including epithelial-mesenchymal transition (EMT), dedifferentiation, tumor-associated cytokines, miRNAs, and hypoxia.

Cancer stemness is influenced by three components: genetic diversity, altered epigenetics, and the tumor microenvironment [34]. The tumor microenvironment is important for cancer cell survival, particularly in solid tumors, because solid tumor cells face challenges during growth, such as hypoxia, low nutrition, and interactions with surrounding normal cells, including tumor-associated fibroblasts, macrophages, the perivascular stroma, and endothelial cells. The tumor microenvironment contributes to CSC maintenance by providing a stem cell niche. Tumor angiogenesis-mediated cancer vascular niche is important for the maintenance and proliferation of CSCs [83]. Stem-like characteristics of BC are not observed until late in tumor development [27]. These findings suggest that the generation of bladder CSCs is a late event in tumorigenesis, and pre-existing BC is likely to supply CSCs via various mechanisms as discussed below (Figure 3).

#### 4.2.1. Epithelial-Mesenchymal Transition (EMT)

The EMT concept emerged from histological findings of embryonic cell development. EMT has been observed in cancer and is an important phenomenon, particularly in tumor invasion and metastasis. Cancer cells originate from epithelial cells; therefore, these cells should initially have many epithelial features. However, during tumor progression, cancer cells may lose their epithelial features and transform into the mesenchymal phenotype. Cancer cells with different phenotypes have been simultaneously observed in a single tumor, including BC [84]. Importantly, Mani *et al.*, demonstrated that breast CSCs are generated via EMT of cancer cells [85,86]. The forced expression of the EMT-inducing transcription factors Twist and Snail in immortalized human mammary epithelial cells results in the acquisition of mesenchymal traits, the expression of stemness markers, and the ability to form spheres [85]. Subsequently, increasing evidence suggests that EMT is a mechanism for generating CSCs. The transforming growth factor β (TGF-β) signaling pathway is involved in both physiological and pathological EMT. The TGF-β pathway is activated in BC and contributes to tumor progression via EMT [87,88,89]. In addition to the TGF-β pathway, hypoxia and its related molecules, hypoxia-inducible factors (HIFs), induce EMT and promote self-renewal in CSCs [90]. The expression of HIF-2α in tumor-associated macrophages is a poor prognostic factor in MIBC [91]. MicroRNAs (miRNAs) are small noncoding RNAs that negatively regulate the expression levels of protein-coding genes and play an important role in various biological processes, including EMT and cancer stemness [92,93]. miRNA-200 regulates EMT in BC, and its expression increases the sensitivity of cancer cells to epidermal growth factor receptor (EGFR) inhibitors [94]. The expression of various other miRNAs are dysregulated in BC, all of which contribute to tumor suppression or promotion [95,96,97].

#### 4.2.2. Tumor-Associated Cytokines

The stemness of breast CSCs is regulated by the cytokine network between mesenchymal stem cells and cancer cells [98]. Plasma IL-6 levels are higher in patients with BC than in healthy controls [99]. Interleukin 6 (IL-6) is a major activator of the Stat3 signaling pathway, which is involved in tumor growth and aggressiveness in various cancers, including BC [100]. In transgenic mice specifically expressing Stat3 in bladder basal cells, a carcinogen, *N*-butyl-*N*-(4-hydroxybutyl)nitrosamine, highly induced bladder premalignant lesions and subsequent invasive BC [55]. BC cells derived from these mice contained a substantial number of CK14^+^ cells, a stem cell marker, and possessed an increased ability to form spheres [55]. Hh signaling is involved in the regulation of stemness in BC [82]. In a murine chemical carcinogenesis model, the loss of Sonic Hh expression in carcinoma *in situ* subsequently reduced local concentrations of bone morphogenetic proteins (BMPs), which are urothelial differentiation factors. Reduced BMP levels promote tumor progression by allowing NMIBC to invade and grow (*i.e.*, progression to MIBC) [82]. Cyclooxygenase-2 (COX-2) is overexpressed in BC compared with the normal urothelium [101], and its major bioproduct, prostaglandins (PGs), are involved in various biological process in BC, including inflammation and carcinogenesis [102]. Nuclear COX-2 is significantly associated with the upregulation of the stemness markers Oct3/4 and CD44v6, suggesting that COX-2 activation is involved in inflammation-mediated bladder CSC proliferation [60].

#### 4.2.3. Cancer Cell Dedifferentiation

When airway tissue is damaged or lost, luminal secretory cells generate multipotent stem cells via dedifferentiation [103]. Dedifferentiation is a possible mechanism of glioblastoma CSC generation [104]. Pathological dedifferentiation is observed in clinical BC, especially in MIBC. Most BC is diagnosed as urothelial carcinoma; however, approximately 10% of urothelial carcinoma contains glandular differentiation, and up to 60% of urothelial carcinoma contains squamous differentiation [105,106]. Moreover, in rare cases, urothelial carcinoma cells can dedifferentiate into other variants, including sarcomatoid, lymphoepithelioma-like, and small cell carcinoma [107]. These pathological variants are usually aggressive and are associated with an unfavorable prognosis [108,109,110]. Interestingly, the gene expression profile of MIBC reveals that squamous or sarcomatoid differentiation is enriched in the basal-typed MIBC, which displays the CSC phenotype [23,108].

#### 4.2.4. Cancer Cell Fusion

Cell fusion may cause cellular transformation as well as cellular diversity, through drastic genotypic changes. Premature chromosome condensation (PCC) is a phenomenon observed when mitotic and interphase cells are fused, and PCC is frequently observed in various cancers, including BC [111,112]. Several mechanisms of cancer cell fusion have been proposed, but oncogenic viruses are the most likely candidate [113]. Human endogenous retroviruses (HERVs) may have oncogenic potential; they interfere with the anti-tumor immune system, promote cancer metastasis, upregulate the expression of oncogenes and growth factors, and inhibit tumor suppressors [114]. The HERV envelope glycoprotein HERVW-1 (also known as syncytin 1) is overexpressed in the placenta and plays an important role in the fusion of placental syncytiotrophoblasts. Additionally, HERVW-1 is overexpressed in certain cancers, such as breast and endometrial cancer [115]. HERVW-1 is reportedly overexpressed in 75.6% of BC tissues, whereas this glycoprotein is overexpressed in 6.1% of matched non-cancer tissues [116]. Interestingly, smoking, the most powerful epidemiological risk factor for bladder carcinogenesis, increases HERV transcription in the urothelium [117]. Apoptosis is another mechanism of cancer cell fusion [70]. The phagocytosis of apoptotic cancer cells may result in the transfer of oncogenes [118], thereby contributing to increased migratory capacity and metastatic potential [119]. Several studies have demonstrated that spontaneous cancer cell fusion promotes cancer aggressiveness and stemness; however, the mechanism of this phenomenon is unclear [120,121,122].

**Figure 4:**
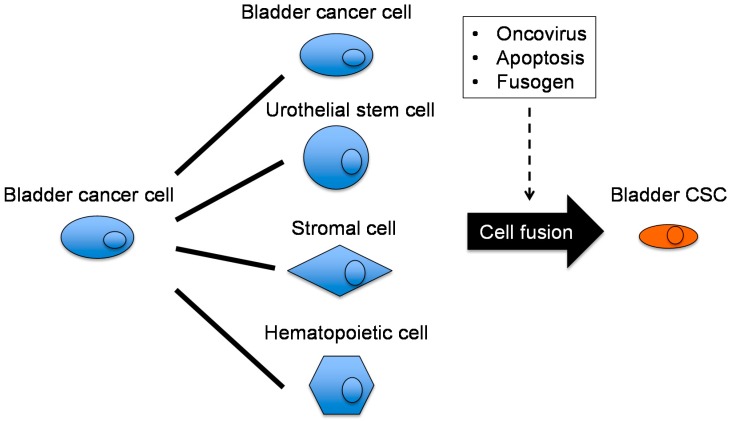
Hypothetical mechanism of cancer cell fusion for bladder CSC generation. Differentiated cancer cells may fuse with various cells, including homophilic fusion with BC cells and heterophilic fusion with urothelial stem cells, stromal cells, and hematopoietic cells. Cell fusion is triggered and driven by some oncoviruses, apoptosis, and fusogens.

Cancer cell fusion results in nuclear reprogramming [123] and is suggested to be a putative mechanism of CSC generation [124]. The generation of bladder CSCs by cancer cell fusion has been suggested [24,125]. Cell fusion is an active biological process and requires fusogens, which are cell fusion proteins that are necessary for the fusion of the cell membrane. Interestingly, a bladder CSC marker, CD44, functions as a fusogen [70,126]. The partners for cell fusion with cancer cells may include various types of stem cells, macrophages [127], and BMDCs [128]. Based on the hypothesis of bladder CSC generation via cell fusion, homophilic (cancer cell to cancer cell) and heterophilic (cancer cell to non-cancer cell) fusion have been proposed (Figure 4).

## 5. Possible Treatments for BC that Target Bladder CSCs

MIBC frequently metastasizes to regional lymph nodes and distant organs. The current standard of care for distal or nodal metastases is cisplatin-based combination chemotherapy. Advanced disease initially exhibits a good response to treatment (an overall response rate of up to 70%); however, most patients develop recurrences and eventually die from the disease [129]. According to the CSC theory, the majority of bladder non-CSCs initially die in response to systematic chemotherapy. However, a small population of bladder CSCs is spared, which results in tumor regrowth and clinical disease recurrence. Recently, several molecular targets for BC have been proposed (*i.e.*, EGFR, FGFR, VEGFR, PI3K-Akt-mTOR, PD-1, COX-2, Aurora kinase A, and several miRNAs), and some chemical compounds are being investigated in clinical trials [130]. A treatment strategy that targets bladder CSCs would be rational and desirable but remains challenging to develop. Several potential therapeutic options for bladder CSCs are discussed below.

### 5.1. Heat Shock Protein 90 (Hsp90) Inhibitors

Hsp90 is a molecular chaperone that is required for the stability and function of numerous oncoproteins that are essential for certain cancer hallmarks, including apoptosis evasion and self-renewal [131]. Hsp90 inhibitors destabilize Hsp90 clients by dissociating Hsp90–Hsp90 client complexes, thereby promoting anti-tumor activity [131]. Hsp90 inhibitors can simultaneously block signaling from multiple oncogenes during the acquisition of therapeutic resistance and self-renewal capacity in glioma CSCs [132] and lymphoma CSCs [133]. Tatokoro *et al.*, demonstrated that Hsp90 inhibitors potentially overcome the cisplatin-resistance of bladder CSCs *in vitro* and *in vivo* [59]. Bladder CSCs, a subpopulation of CD44^+^ BC cells, exhibited higher activity in the Akt and Erk signaling pathways compared with their CD44^−^ counterparts. An Hsp90 inhibitor, 17-DMAG, simultaneously inactivated both the Akt and Erk signaling pathways at non-cytotoxic concentrations and synergistically potentiated the cytotoxicity of cisplatin against bladder CSCs by enhancing apoptosis [59]. A potent Hsp90 inhibitor, ganetespib, is now being used in clinical trials and is expected to overcome the therapeutic resistance of CSCs in a wide variety of malignancies, including BC [134].

### 5.2. Blocking Cyclooxygenase-2 (COX-2)-Prostaglandin-E2 (PGE2) Signaling

Chemoresistance is a major challenge in the treatment of MIBC. During wound repair, normal stem cells are recruited from a quiescent pool at the wound site [135]. Similarly, bladder CSCs are mobilized to regrow in response to chemotherapy-induced damage, which contributes to the chemotherapy resistance of BC. Kurtova *et al.*, found that chemotherapy releases PGE2 while effectively inducing apoptosis in human BC xenografts, which promotes CSC repopulation and tumor regrowth between chemotherapy cycles [136]. A PGE2-neutralizing antibody and the COX-2 inhibitor celecoxib, which inhibits PGE2 production, abrogate CSC repopulation during chemotherapy intervals and effectively attenuate the progressive manifestation of chemoresistance [136].

### 5.3. Blockade of CD47

Intravesical instillation of bacillus Calmette-Guerin (BCG) has long been the standard of care for high-risk NMIBC, whereas its effectiveness against MIBC is limited. Macrophages play a key role in antigen presentation to T-cells via phagocytosis to elicit an anti-tumor immune response [137]. Recent clinical studies have demonstrated that immune checkpoint inhibition, which restores anti-tumor T-cell immunity, is associated with a beneficial objective response and remarkably improves the prognosis of patients with advanced malignancies, including patients with BC [138]. Chan *et al.* reported another potential type of immunotherapy against BC, where bladder CSCs express higher levels of CD47, a cell surface protein that provides an inhibitory signal for macrophage phagocytosis, compared with the rest of the tumor [20,139]. Blockade of CD47 with a monoclonal antibody resulted in macrophage engulfment of BC cells *in vitro*, suggesting that immunotherapy against CD47 could be effective in the treatment of MIBC.

### 5.4. Telomerase Inhibitors

Telomerase activity is up-regulated in 85% to 90% of cancers [140]. Telomerase is activated in a vast majority of tumor cells and in CSCs [141], and the telomere length of CSCs is shorter than that of the majority of tumor cells [142,143]. The enforced elongation of the telomere length of cancer cells induces cellular differentiation, which suggests that telomere length is related to cancer cell differentiation [144]. Of particular note is that CSCs are suggested to be sensitive to telomerase-based therapy [145]. TERT promoter mutations and subsequent telomerase reactivation is often observed in BC [146]. The C228T mutation of the TERT promoter frequently occurs in bladder CSCs, which contributes to tumorigenesis [147]. Although not specifically in bladder CSCs, the telomerase inhibitor GRN163L induces growth arrest in T24 BC cells but not in normal urothelial cells [148]. Furthermore, antisense oligodeoxynucleotides against the telomerase enzyme hTERT reduced the growth of BC cells [149]. These findings suggest that telomerase inhibitors may be promising agents for bladder CSCs.

### 5.5. Retinoids

Retinoids are signaling molecules that regulate cellular events, such as development, differentiation, proliferation, and apoptosis, by binding to RAR/RXR receptors, which initiates a cascade of changes in the structure of chromatin [150]. Retinoids have been shown to regulate self-renewal and pluripotency in ES and progenitor cells [151] and to promote differentiation and initiate stable epigenetic changes in CSCs [152]. All-trans retinoic acid (ATRA) is a powerful antineoplastic agent for human acute promyelocytic leukemia (APL) [153]. ATRA exerts anti-tumor effects on glioma CSCs [154] and lung CSCs [155] via differentiation and a reversion to heterogeneity. In addition, retinoid signaling is involved in the specification and regeneration of urothelial progenitor basal cells [156]. In BC, retinoic acid inhibits both VEGF and EGF signaling, thereby reducing the recurrence rate and improving the prognosis of NMIBC patients [157].

### 5.6. Other Putative Strategies for Targeting Bladder CSCs

CSCs use the same signaling pathway as ES cells. Therefore, the Wnt, Notch, and Hh pathways are emerging as therapeutic targets for CSCs [158]. Wnt signaling is activated and contributes to cancer progression in BC [49,159]; however, the therapeutic efficacy of Wnt signaling inhibition in bladder CSCs is not well studied. The G protein-coupled receptor smoothened (SMO) is a downstream effector of the Hh pathway, and the selective SMO antagonist LDE225 is now being studied in clinical trials of BC [130]. Reportedly, TGF-β signaling maintains the cancer stemness of glioma cells by up-regulating SOX2 [160]. Plasma TGF-β1 levels are elevated in patients with MIBC [161], and a TGF-β receptor inhibitor attenuates the invasive potential of BC cells [162], which suggests that inhibition of TGF-β signaling may be effective in abrogating the stemness of BC. However, TGF-β initially inhibits epithelial growth [163]; therefore, TGF-β inhibitors should be used with caution in cancer patients. B-cell-specific Moloney murine leukemia virus insertion site 1 (Bmi1) plays a role in self-renewal and differentiation in both normal stem cells and CSCs. A small molecule inhibitor for Bmi1, PTC-209, inhibits tumor growth in colon cancer xenografts with long-term and irreversible effects [164]. In bladder CSCs, Bmi1 knockdown impairs cell proliferation, migration, and sphere formation, and enhances the sensitivity of cells to cisplatin treatment [165].

## 6. Future Perspectives on Bladder CSC Research

The CSC theory was first established in human blood malignancies as a model for normal hematopoiesis and has since been progressively applied to many solid tumors. Cultured cancer cell lines derived from solid tumors in patients and genetically engineered mice have increased our understanding of CSC cancer biology; however, traditional CSC models have not predicted clinical success [5]. Several questions and controversies regarding CSCs in solid tumors need to be addressed. Most cancer cell lines derived from heterogeneous solid tumors are selected for plastic culture systems with a nutrition and oxygen-rich environment; however, the xenograft tumors used to define CSCs exhibit cellular homogeneity and do not mimic solid tumors with tumor heterogeneity. This finding suggests that established cancer cell lines predominantly or exclusively have self-renewal potential rather than differentiation potential, which indicates that they may be almost identical to CSCs without isolation. Therefore, isolated cells that express various markers may be highly reproducible or self-renewing cancer cells. Another concern is that CSCs in solid tumors are usually isolated after dissociation into single cells; however, in contrast to blood malignancies, tumorigenicity requires cell-cell or cell-matrix attachment by cancer cells and an assembly of stromal cells, such as endothelial cells, fibroblasts, and inflammatory cells. The hypotheses on the origin of CSCs, the underlying mechanism of CSC generation, and the existence of CSCs in solid tumors in the human body are inconsistent and inconclusive. However we may already have clues to answer these questions, because we have repeatedly observed a small number of living tumor cells as well as remarkable tumor shrinkage, by antineoplastic agents in *in vivo* experiments and in clinical trials.

In the 21st century, most types of cancer, including BC, are still incurable when they metastasize. The hypothesis of bladder CSCs is strongly supported by recent studies; however, we have not yet obtained conclusive evidence of the existence of bladder CSCs in the clinical setting. It is urgent that we identify specific markers for bladder CSCs and demonstrate both the pluripotency and self-renewal potential of bladder CSCs derived from clinical samples. As described in this review, bladder CSCs may be generated and maintained by more dynamic biological processes than initially expected. Bladder CSCs may inevitably emerge in the residual tumor via an unknown dynamic mechanism. Accumulating evidence of cancer cell plasticity suggests that therapies targeting either bladder CSCs or bladder non-CSCs are not sufficient to achieve complete recovery. A plausible and attractive strategy may be to regain the organizing ability of the BC cells and shift them to a normal differentiation process. We believe that a better understanding of bladder CSC biology may improve the therapeutic outcomes of BC in the near future.
